# Intimate partner violence during the COVID-19 pandemic in Western and Southern European countries

**DOI:** 10.1093/eurpub/ckab093

**Published:** 2021-08-18

**Authors:** Julia Brink, Patricia Cullen, Kristen Beek, Sanne A E Peters

**Affiliations:** 1 Julius Center for Health Sciences and Primary Care, University Medical Center Utrecht, Utrecht University, Utrecht, The Netherlands; 2 School of Population Health, UNSW, Sydney, Australia; 3 The George Institute for Global Health, UNSW, Sydney, Australia; 4 Ngarruwan Ngadju: First Peoples Health and Wellbeing Research Centre, University of Wollongong, Wollongong, Australia; 5 The George Institute for Global Health, Imperial College London, London, UK

## Abstract

**Background:**

Intimate partner violence (IPV) is a significant problem with several negative health outcomes. Disasters are linked to increased IPV, but little is known about reporting of and strategies to address IPV during the COVID-19 pandemic. This review maps the IPV reporting during the pandemic and interventions to prevent and respond to IPV in 11 Western and Southern European countries.

**Methods:**

Government websites, news articles and pre-prints were searched using the terms ‘domestic violence’ or ‘violence’ in combination with ‘Covid’ or ‘Corona’. Embase, PubMed, Scopus and Google Scholar were searched using the terms ‘domestic violence’ and ‘partner violence’ and ‘interventions’.

**Results:**

Six countries showed an increase in domestic violence reports (Austria, Belgium, France, Ireland, Spain and UK), two countries a drop (Italy and Portugal), two countries showed no change (The Netherlands and Switzerland) and one country did not provide comparative data (Germany). Common measures to address IPV were starting a campaign (nine countries), creating online support (seven), more funding for alternative accommodation (seven) and support (eight) and use of a code word (four).

**Conclusions:**

IPV reports or helpline calls in Western and Southern European countries in the first weeks of COVID-19 measures increased in six countries, remained the same in two countries and showed a decrease in two countries. While this review cannot ascertain the impact of the measures taken by the countries during the pandemic and beyond, this mapping provides a foundation for future research, and an opportunity to trace the efficacy of these strategies.

## Introduction

Globally, one in three women have experienced physical and/or sexual violence by an intimate partner, or sexual violence in their lifetime.^1^ The most common form of violence against women is domestic violence, and more specifically domestic violence perpetrated by an intimate partner, called intimate partner violence (IPV).^1^^,^^2^ In Western Europe, 19.3% of ever-partnered woman has experienced IPV in their lifetime.^1^ IPV can be physical, sexual, emotional (including coercive and controlling behaviour), and can also include economic or financial control.^3^ Although IPV can be committed by women, the most common perpetrators are male intimate partners or ex-partners.^4^ IPV has long-term impacts on physical and psychosocial health and has been linked to increased health risk behaviours.^5–10^ There are also profound consequences for children who witness IPV, with higher rates of behaviour and psychological problems, difficulties at school and increased likelihood that they will experience or perpetrate IPV as adults.^11–13^

Crises and disasters have been linked to increased IPV. Since the COVID-19 pandemic can be regarded as a crisis that shows similarities to economic crises and natural disasters, there is widespread concern about increases in IPV during the pandemic.^14–16^ The United Nations expect an additional 15 million cases of gender-based violence for every 3 months of lockdown.^17^ A diplomatic statement made by 56 countries underscored that ‘participation, protection and potential of all women and girls must be at the centre of response efforts’ during the COVID-19 pandemic. It also acknowledges that restrictive measures increase the risk of domestic violence, and assert that specific measures should be implemented to prevent violence against women and girls.^18^

Notwithstanding the known gendered drivers of IPV, the reasons for increased IPV during COVID-19 are broadly 3-fold. First, pandemics, health emergencies and quarantine periods are associated with negative emotions, problematic coping and mental health disorders.^19–22^ For instance, alcohol consumption has increased in the lockdown period as a coping mechanism for boredom, increased stress and other mental health problems.^23^^,^^24^ Secondly, social distancing leads to an increase in exposure to perpetrators, less support opportunities for both survivors and perpetrators and facilitates perpetrator tactics of isolation and control. Thirdly, the pandemic leads to more financial distress and unemployment, which are both associated with an increased likelihood of IPV.^12^^,^^25^ Economic uncertainty and anticipatory anxiety, apart from the actual financial hardship, also leads to a rise in IPV.^25^ Women are also less likely to leave the abusive partner when they are financially dependent.^26^

Although there has been much media attention on the impact of COVID-19 on IPV, scant literature is available that shows if and to what extent IPV has increased during the pandemic, and what country-level policies are in place to address this violence. Therefore, to address this, we systematically mapped the reported IPV in Western and Southern European Countries since the onset of the pandemic and identified actions that have been taken to prevent and respond to IPV.

## Methods

Countries included in this review are the UK, The Netherlands, France, Italy, Spain, Flanders, Germany, Austria, Switzerland, Ireland and Portugal. These countries are included because of their geographic proximity and because they are, at a global scale, relatively comparable in terms of human development and gender inequality.^27^ References in the result section are added to a Supplementary, given the large number of references.

### Search strategy

PubMed, Scopus, Embase and Google scholar were searched in June 2020. As this resulted in limited information, we consulted the websites of the national governments and of the countries’ main charity for domestic violence and partner violence for country-specific information on the change in IPV during the pandemic and mitigation measures. Data from pre-print articles found via Medrvix were identified using the terms ‘domestic violence’, ‘intimate partner violence’ or ‘violence against woman’ in combination with the name of a country and ‘Covid’ or ‘Corona’, eventually in combination with ‘data’ or ‘prevalence’. Additionally, major media and newspapers were used as resources. The identified country-specific resources are listed in Supplementary table S1. All searches were done both in English and in the main language of the country, using a translation tool.^28^ The websites of the main organizations that respond to domestic, family and IPV and violence against women in the specific countries were also searched. Literature on possible interventions to mitigate IPV was collected by searching via Embase, PubMed, Scopus and Google scholar for reviews on ‘domestic violence’, ‘partner violence’ and ‘interventions’. References of selected articles were also checked to identify any additional articles.

### Data extraction and mapping

**Figure 1:**
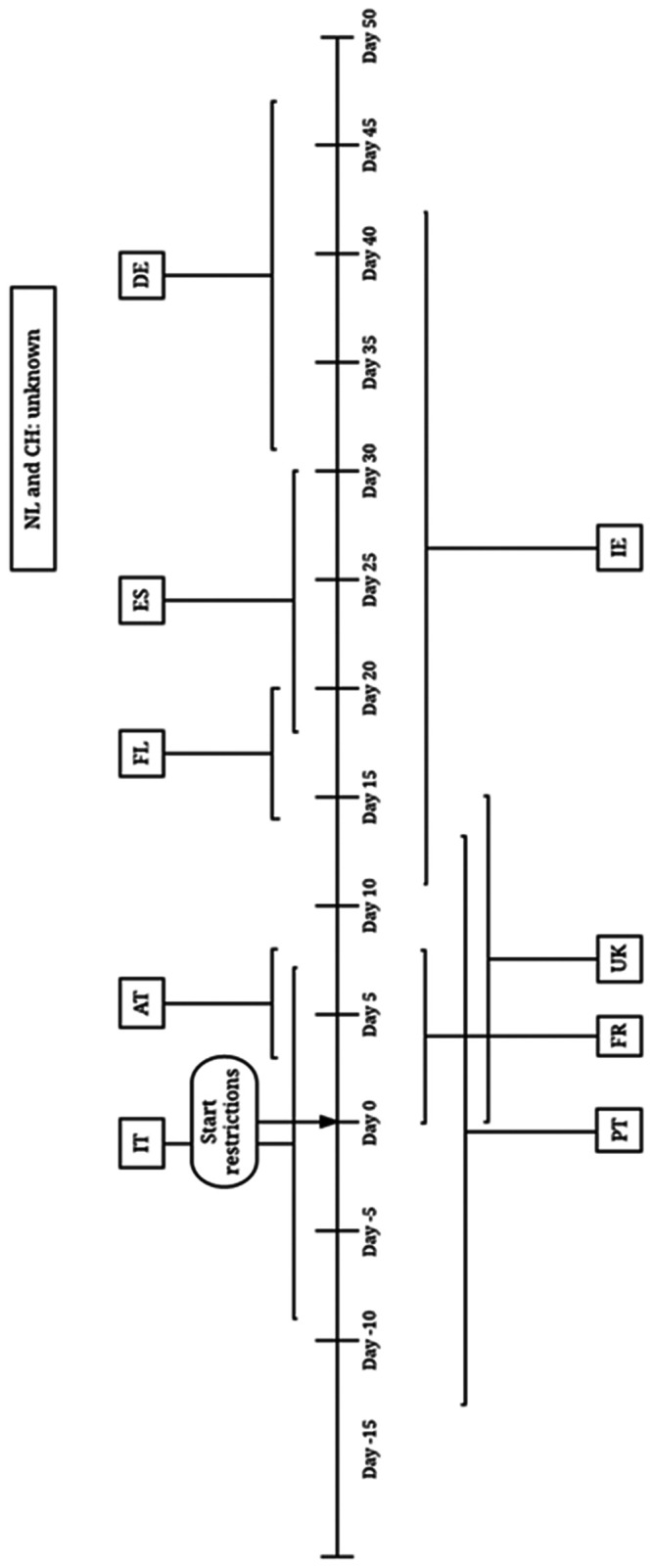
Timeline showing the period in which data on domestic violence was collected compared to the start of the movement restrictions AT, Austria; FL, Flanders; FR, France; DE, Germany; IE, Ireland; IT, Italy; PT, Portugal; ES, Spain; CH, Switzerland; NL, The Netherlands; UK, United Kingdom. Start of the stay at home phase for the different countries was; Austria, March 16; Flanders, March 17; France, March 17; Germany, March 22; Ireland, March 27; Italy, March 10; Portugal, March 18; Spain, March 15; Switzerland, March 16; The Netherlands, March 23; UK, March 26.

Data on reported IPV and intervention taken were extracted at country-level using a tailor-made data collection sheet. Changes in reported IPV were mapped against the commencement of highest level of restrictions to provide a country-specific overview of data on changes in rates of reported IPV. Figure 1 shows the timeline in which data were collected, compared to the commencement of stay at home public health orders in each of the countries. The restrictions are classified as high level, moderate and low level according to the extent of the stay at home public health orders in place.

## Results

### IPV in Western and Southern Europe during COVID-19

Stay at home public health orders came into effect in Italy on 10 March 2020, and were subsequently implemented across the remaining 10 countries, with Ireland being the last to implement restrictions on 27 March 2020. The restrictions were variable in their intensity, with one country (Italy) implementing high-intensity restrictions. By contrast, five countries, Germany, Spain, Switzerland, The Netherlands and UK, implemented relatively low-intensity restrictions, and the remaining five countries implemented orders that were considered to be moderate-intensity restrictions (table 1). For classifying the intensity of the restrictions, ‘Oxford COVID-19 Government Response Tracker’ is used.^29^

**Table 1:** Domestic violence during the COVID-19 restrictions per country

Intensity of restrictions	85	81	88	77	85
**Increase in IPV reports**	++	++	+	?	+
**Online survey**				April 22–May 8: per cent of questioned woman Physical violence: 3.1% Sexual violence: 3.6% Emotional violence: 3.8% felt threatened, 2.2% was forbidden to leave the house, 4.6% was controlled in contacts with others	
**Police reports**			March 17–24: a rise of 32% at rural and suburban area’s March 17–26: a rise of 36% in Paris		2020: a rise of 25% compared to 2019
**Helpline calls**	March 19–24: 57% rise	March 27–April 3: a rise of 70% in the third week of the restrictions comparing to the first week			March 23–May 24: a rise of 39% compared to the same period in 2019
**Country**	Austria	Flanders	France	Germany	Ireland


**Strictness of measures**	92	82	72	73	74	76

**Increase in IPV**	—	−	++	No change	No change	++
**Online survey**						
**Police reports**	March 1–22: a drop of 44% comparing to the same period in 2019	March: GNR reports a drop of 26%, the PSP a drop of 15%	Sharp drop	No significant rise or drop	No significant rise or drop	
**Helpline calls**	March 1–14: a drop of 55% comparing to the same period in 2019		April 1– 14: 47% increase	No significant rise or drop	No significant rise or drop	March 26–April 6: a rise of 25% comparing to the average before the pandemic March 26–April 13: 49% rise comparing to the average before the pandemic
**Country**	Italy	Portugal	Spain	Switzerland	The Netherlands	UK


++, >40% rise; +, <40% rise; ±, no changes; −, <40% drop; —, >40% drop.

70–79, relatively low-intensity restrictions; 80–89, relatively moderate-intensity restrictions; 90–100, relatively high-intensity restrictions.

Total of 6 out of 11 countries showed an increase in reported IPV, 2 reported no change, 2 reported a decrease and 1 provided no comparative data. Of the six countries showing an increase, four (Austria, Flanders, Spain and UK) reported an increase of more than 40%, indicated by an increase in calls to helplines. Of these, four had moderate-intensity restrictions in place and Spain and UK had low-intensity restrictions. In contrast to increased helpline calls, there was a sharp decrease in police reports of IPV in Spain during this time. Likewise, in Italy, there was a decrease in police reports compared with the same period in 2019, but also in helpline calls.

Helpline data were available for eight countries, and five countries (Austria, Flanders, Ireland, Spain and UK), all of which three had high to moderate-intensity restrictions and two had low-intensity restrictions, reported increased calls. Police report data were available for seven countries, with increased IPV evident in two countries (France and Ireland), which each had moderate-intensity restrictions in place. Of the remaining five countries with police data, three showed a decrease in reports of IPV, and of these, one country had high-intensity level restrictions in place (Italy).

Only Germany collected data from women, via an online survey investigating experiences of domestic violence during the restrictions among women aged 18–65 years (*n*=3800). The survey indicated that 3.1% of women was involved in at least one form of physical conflict in the home; 3.6% of women was forced into sex by their partners; 3.8% felt threatened by their partners; 2.2% were not permitted to leave home without permission; and 4.6% of partners controlled the woman’s contact with others, including digital channels, such as messenger services. This data cannot be compared to pre-pandemic data since past studies surveyed experiences of violence over longer periods, not after a period of only a few weeks; thereby it was not clear if there were changes to IPV prevalence.^30^

### Actions taken to mitigate the burden of IPV during COVID-19

The countries have implemented various measures to prevent and respond to IPV since the onset of the pandemic, which are outlined by country (table 2). Some of the measures refer more broadly to domestic violence, which also encompasses IPV, and are hence reported.

**Table 2:** Measures taken by countries to reduce the impact and prevalence of IPV during COVID-19

	AT	BE	FR	DE	IE	IT	PT	ES	CH	NL	UK
Campaign and information brochures for survivors	X	X			X	X	X	X	X	X	X
Online support in addition to existing helplines		X		X		X	X	X		X	X
Extra funding for alternative accommodation services	X	X				X		X	X	X	X
Extra funding for support services	X	X	X		X	X	X	X			X
Code word to connect with service providers		X	X					X		X	
Specifically mention it that victims are exempted from restrictions if in danger		X		X	X						
Advice brochures for health workers										X	
Protective rules for survivors, such as					X	X					X
Extra focus on arresting and prosecuting of perpetrators					X						
Police making proactive contact with people deemed to be at risk of IPV					X						
Guidelines for health professionals										X	
Task Force to observe									X		
Symbol of hope campaign for survivors											X

AT, Austria; BE, Belgium; FR, France; DE, Germany; IE, Ireland; IT, Italy; PT, Portugal; ES, Spain; CH, Switzerland; NL, The Netherlands; UK, United Kingdom.

#### Austria

On March 19, the Minister of Women and Minister of Justice presented a package of measures to protect women from domestic violence. This package contained extra financial resources and extra personnel for domestic violence helplines, the possibility of online counselling, information brochures at supermarkets and extra alternative accommodations.

#### Belgium

In response to increased domestic violence, the Belgian government granted additional funding to the national helpline, which was used to create an online chat function, to expand the capacity and the opening hours of the helpline, and to launch a poster campaign focussing on preventing domestic violence and encouraging help seeking. Additional funding was directed towards education on trauma counselling for psychologists and to ensure the availability of alternative accommodation. In terms of the restrictions, people were permitted to leave their house if escaping risk of harm and/or seeking help. Furthermore, in Flanders, the code word ‘Masker-19’ could be used in pharmacies by those seeking help for domestic violence.

#### France

The French government established new facilities for women seeking support, investing 1 million euros in help centres. The priority was to ensure survivors were safe at home, by providing alternative accommodations for perpetrators, including special centres and prison, as well as the launch of a special platform to find alternative housing as quickly as possible. Furthermore, a hotline was launched to assist perpetrators in anger management and, if needed, to access alternative accommodation. The existing helpline for women who are survivors of IPV and domestic violence was expanded. The code word ‘Masque-19’ could be used in pharmacies.

#### Germany

The Federal Minister prioritized ensuring shelters for those escaping IPV remained open, and confirmed intention to expand to digital and telephone hotlines. People were permitted to leave their house if escaping risk of harm and/or seeking help. Local authorities were responsible for financing alternative accommodations.

#### Ireland

Operation Faoiseamh commenced on April 1, with the first phase involving police making proactive contact with those who had reported IPV in the past and actively following-up. The second phase concentrated on the arrest and the prosecution of perpetrators. Additionally, the Government and frontline organizations launched the Still Here campaign to create awareness for IPV and reassure that support remained available. People were permitted to leave their house if escaping risk of harm and/or seeking help. In June, a rent supplement was made more readily accessible for survivors of domestic violence, which provided a rent supplement for at least 3 months for alternative housing.

#### Italy

On April 2, the Italian government provided an extra 30 million euro for anti-violence foundations, which was directed towards shelters and funding for initiatives to support survivors. The government also launched a new application that enabled people to access help without making a phone call. Survivors could access information brochures and support at pharmacies. Prosecutors also ruled that the perpetrator, rather than the victim/survivor, must leave the home in situations of domestic violence.

#### Portugal

The government expanded the capacity of assistance for survivors by creating a special e-mail address and SMS-helpline, expanding staff in two shelters and enhancing contact with support groups. A campaign was launched to create more awareness of domestic violence.

#### Spain

Shortly after the lockdown, a ‘Contingency Plan Against Gender-based Violence’ was adopted by the government to minimize the risk of violence against women and girls resulting from confinement measures. This plan contained four measures. Firstly, specialized services that protect and assist women and children were declared as essential, including a 24-h helpline, ‘ATENPRO Service’ and online services monitoring compliance with restraining orders and alternative housing for women. Secondly, a new instant messaging service via WhatsApp, available 24 h a day, for counselling by female psychologists was implemented. Thirdly, a prevention and awareness campaign against gendered violence during the confinement period was launched. Lastly, a guide for women experiencing gendered violence, containing a list of questions, procedures and information, was published. Additionally, the Canary Islands in Spain were the first to introduce a code word ‘Mascarilla-19’ for people to access support at pharmacies.

#### Switzerland

Although no increase in domestic violence was reported in Switzerland, the government was concerned about potential increases and a task force was created to monitor domestic violence. The task force functioned as an interface between authorities and launched a campaign to create awareness for violence and to promote help seeking. Some regions expanded their capacities for support and alternative accommodation.

#### The Netherlands

The government started a campaign to mitigate domestic violence on April 25, which focussed on giving citizens concrete advice on how they could help when they suspect a form of domestic violence. Furthermore, the government created guidelines for health professionals treating patients that were or may have been at risk of domestic violence. The organization ‘Veilig Thuis [Safe at home]’ started an anonymous chat function on May 25. The code word ‘Masker-19’ could be used in pharmacies.

#### UK

The government of the UK allocated 750 million pounds to charities including those providing domestic violence services, and an extra 2 billion pounds was made available to enhance online support services and helplines for domestic abuse and creating alternative accommodation. A new national communications campaign was launched to reach out to those at risk of abuse. Also, a symbol of hope, a handprint with a heart on it, was created to show solidarity to those impacted by domestic abuse. Furthermore, the government progressed the Domestic Abuse Bill, which included a new duty on local authorities to assess the need and commission support to survivors and their children in safe accommodation. The government also planned to bring forward legislation so that those fleeing domestic abuse and facing homelessness would be automatically considered as a priority need by their council for settled housing.

## Discussion

This review on the impact of the COVID-19 pandemic on IPV in Western and Southern European countries indicates considerable variability in IPV trends, with five countries demonstrating an increase in reported IPV, two countries a decrease, two countries showed no change and one country was unclear. A diverse range of measures has been undertaken to mitigate the impact of COVID-19 on IPV, ranging from campaigns, online support, using a code word to connect with service providers and increased funding for alternative accommodation and survivor support. Despite some commonalities in restrictions implemented to curb the spread of COVID-19, this review demonstrates that in counties across Western and Southern Europe there is considerable variation in IPV, reporting and responses. These findings have important implications for policy and service responses to prevent and respond to IPV during and beyond the pandemic.

In our review, four of the six countries reporting an increase in IPV had moderate to high-intensity restrictions, indicating that, as anticipated, the restrictions may be driving increases in IPV. However, high-intensity restrictions can also make it more difficult to report violence and seek help, which may explain why in our review, despite high-intensity restrictions, Italy did not report increases in IPV. Thereby, potentially, in countries with high-intensity restrictions that forbid leaving the house for non-essential purposes, there may be a greater increase of IPV due to increased exposure to perpetrators and fewer opportunities for accessing support than in countries where restrictions are less intensive.

To varying degrees, increases in IPV, and domestic violence more broadly, during the COVID-19 pandemic have also been reported in other regions, including Australia,^31^ Brazil,^32^ China,^33^ India^34^ and USA.^35^ Taken together with our results, this suggests that regardless of region and culture, the COVID-19 pandemic is impacting the prevalence of IPV globally, however, there are substantial issues in measurement of IPV due to possible under-reporting and reduced help seeking during the pandemic, particularly for non-physical forms of violence and abuse.

Although the data on IPV during the pandemic is not fully comparable between countries due to differences in measurements methods, it is important to consider contributing factors to explain disparity in IPV trends during the pandemic. A possible explanation for differences in IPV trends relates to existing interventions for both perpetrators and survivors that were in place before the pandemic. Also, as gender inequality at country-level is a key driver of violence against women, differences in gender equality may be another possible explanation for disparity between countries.^36^ Although gender inequality serves as a driver for IPV, interpretation of the link between gender equality and IPV reports may be complicated when a higher level of gender equality might increase the likelihood that women report violence.^37^

This review revealed a number of strategies applied during the COVID-19 pandemic to prevent IPV and support survivors. The most widely implemented of these was the initiation of a campaign and/or brochure to provide information to survivors of IPV. This strategy was applied in 9 of the 11 countries. Following this, extra funding for the support services for survivors was provided in eight countries, while seven countries each implemented online support services to complement existing helplines, or increased capacity to provide alternative accommodation. Various interventions centred on protective rules (three countries), arrest and prosecution of perpetrators (one country) and proactive outreach policing (one country) were also mapped across the region. The innovative approach of introducing a standard code word for use at pharmacies was found in four countries. Campaigning to raise awareness of domestic violence and show support for survivors was seen in one context, as was the development of an advice brochure for health workers.

Taken together, these varied interventions both supplemented existing measures and attempted to address the specific risks and challenges brought forward by the pandemic. Mapping what was done in relation to IPV during the COVID-19 pandemic is an important first step to understanding feasibility and effectiveness of interventions during a pandemic, for scale up and implementation post-pandemic and to build systems of prevention and response to IPV that will withstand future shocks. To our knowledge, this review is the first comprehensive overview of IPV and response measures during the COVID-19 pandemic in Western and Southern European countries. It provides insight into the current situation and the relationship between pandemic-related restrictions and IPV. This review provides insight into interventions that have been implemented to address IPV during the pandemic and may therefore contribute to retrospective research that evaluates the impact of such interventions. These studies would be valuable in strengthening policy responses to IPV both during the pandemic and beyond.

There are limitations to this review. Firstly, data on IPV during the pandemic cannot be compared between countries due to variation in data sources, periods of measurement, comparison values and the possibility of under-reporting. Also, different biases might arise from the differences in data collection methods, such as selection in online surveys and delays in reporting in registries. However, within data sources, the data collection methods were the same before and during the pandemic. Further, the prevalence IPV reports during the pandemic is compared with different reference periods, varying from the prevalence in the previous month, to the same period the previous year, the whole year 2019, and the first week of restrictions. Also, the degree of under-reporting of IPV in the different countries remains unknown and may vary between countries. Under-reporting might be an explanation for the decrease in IPV reports in Italy and Portugal, but this has yet to be investigated. Furthermore, some data emanates from news articles rather than official reports. Data on the measures that are taken by countries emanates from government websites and news articles, which may be incomplete. Lastly, no data on IPV was available after measures were taken to reduce IPV during the pandemic, thereby the effect of the interventions remains unknown. Once data on IPV prevalence after the measures were implemented is available, analyses of the effect of the interventions could be undertaken. This is an important focus for future research, since there is limited insight into the effectiveness of interventions in comparable situations, due to the lack of recent, similar epidemics in Europe.

In conclusion, early data suggest that the number of IPV reports or helpline calls in Western and Southern European countries in the first weeks of COVID-19 measures increased in six countries, remained the same in two countries, and showed a decrease in two countries. Measures taken to mitigate the impact of COVID-19 on IPV by multiple countries include increasing capacity for alternative accommodation, additional funding for support services, initiating campaigns to raise awareness, implementing a code word system and creating additional means of communication, such as online chat functions. While it is not possible from this review to ascertain the impact of these measures on the prevalence of IPV in selected countries during the pandemic and beyond, this mapping provides an early foundation for future research, and an opportunity to trace the varied efficacy of these strategies. The effectiveness of any crisis response is dependent on policies and practices in place before the disaster strikes. Understanding what has been done, and eventually how this has impacted IPV will enable evidence-informed preparedness activities across sectors and organizations involved in supporting survivors of IPV during periods of increased risk, including for future pandemics and other adverse events.

## Supplementary data

Supplementary data are available at *EURPUB* online.

## Funding

This study was supported by a COVID-19 research grant from the University of New South Wales. P.C. is funded by a National Health and Medical Research Council Early Career Fellowship (Grant ID: APP1158223). S.A.E.P. was supported by a UK Medical Research Council Skills Development Fellowship (MR/P014550/1).

*Conflicts of interest*: None declared.

Key points

- IPV reports or helpline calls in Western and Southern European countries in the first weeks of COVID-19 measures increased in six countries, remained the same in two countries and showed a decrease in two countries.
- The most common measures to reduce the impact of IPV taken were starting a campaign, creating online support, creating more funding for alternative accommodation and support and use of a code word.
- This review provides an important foundation for tracking IPV during the pandemic and an opportunity to trace the varied efficacy of these strategies.

## Supplementary Material

ckab093_Supplementary_Data
