# Supplementary material for: Intimate partner violence during the COVID-19 pandemic in Western and Southern European countries
Source: Eur J Public Health. 2021 Aug 18;31(5):1058–63. doi: 10.1093/eurpub/ckab093 (PMC8436372; doi:10.1093/eurpub/ckab093)
Supplement: ckab093_Supplementary_Data [file ckab093_supplementary_data.docx]

**Supplementary table: Country-specific resources used**

| **Resources** |
| --- |
| **Austria**   - Was nun erlaubt ist und was nicht [Internet]. ORF news. [cited 2020 Jun 19]. Available from: [https://orf.at/stories/3158055/](NULL) - Coronavirus: Leichter Anstieg bei häuslicher Gewalt [Internet]. Vienna. [cited 2020 Jun 16]. Available from: [https://www.vienna.at/coronavirus-leichter-anstieg-bei-haeuslicher-gewalt/6578962](NULL) - Maßnahmenpaket gegen häusliche Gewalt [Internet]. ORF. [cited 2020 Jun 16]. Available from: [https://orf.at/stories/3158536/](NULL) - Raab: Offensive gegen häusliche Gewalt [Internet]. Bundeskanzleramt. [cited 2020 Jun 16]. Available from: [https://www.bundeskanzleramt.gv.at/bundeskanzleramt/nachrichten-der-bundesregierung/2020/raab-offensive-gegen-haeusliche-gewalt-.html](NULL) |
| **Belgium/Flanders**   - Coronavirus: Phase 2 maintained, transition to the federal phase and assitional measures [Internet]. Info-coronavirus.be. [cited 2020 Jun 19]. Available from: [https://www.info-coronavirus.be/en/news/phase-2-maintained-transition-to-the-federal-phase-and-additional-measures/](NULL) - Coronavirus: reinforced measures [Internet]. Belgium.be. [cited 2020 Jun 19]. Available from: [https://www.belgium.be/en/news/2020/coronavirus_reinforced_measures](NULL) - Gecoördineerde steun voor vrouwen die verzwakt zijn door de coronacrisis [Internet]. CDV. [cited 2020 Jun 15]. Available from: [http://www.wouterbeke.be/nieuws/gecoordineerde-steun-voor-vrouwen-die-verzwakt-zijn-door-de-coronacrisis/](NULL) - Belga. Zeventig procent meer oproepen over geweld bij hulplijn 1712 sinds lockdown [Internet]. HLN. [cited 2020 Jun 15]. Available from: [https://www.hln.be/nieuws/zeventig-procent-meer-oproepen-over-geweld-bij-hulplijn-1712-sinds-lockdown~a669952e/?referer=https%3A%2F%2Fen.wikipedia.org%2F](NULL) - Van Hoofstat J. Kan ‘masker 19’ helpen in strijd tegen huiselijk geweld tijdens coronacrisis? Niet iedereen is enthousiast [Internet]. Het Nieuwsblad. [cited 2020 Jun 15]. Available from: [https://www.nieuwsblad.be/cnt/dmf20200407_04915813?articlehash=DDBC902C2B81C1C7D8AE10B31B3EA3FD227907F4315BED25DB31C5B313E5DF9CF2973F85CAA96D51C29C7A2DED4ECD20A2DFBC8773B81F25E6AF57C960020130](NULL) - 5.409 OPROEPEN OVER 7.402 MENSEN VOOR HULPLIJN 1712 IN 2019. [Internet]. 1712. [cited 2020 Jun 20]. Available from: [https://1712.be/archief/nieuws/id/662/5409-oproepen-over-7402-mensen-voor-hulplijn-1712-in-2019](NULL) - Meesters J. Slachtoffer van huiselijk geweld? Noem codewoord ‘masker 19’ in de apotheek [Internet]. Hbvl. [cited 2020 Jun 15]. Available from: [https://www.hbvl.be/cnt/dmf20200413_04921435/slachtoffer-van-huiselijk-geweld-noem-codewoord-masker-19-in-de-apotheek](NULL) |
| **France**   - Emmanuel Macron annonce l’interdiction des déplacements non essentiels dès mardi midi [Internet]. Magazine Marianne. [cited 2020 Jun 19]. Available from: [https://www.marianne.net/politique/emmanuel-macron-annonce-l-interdiction-des-deplacements-non-essentiels-des-mardi-midi](NULL) - Domestic violence increases in France during Covid-19 lockdown [Internet]. Euractiv Network. [cited 2020 Jun 11]. Available from: [https://www.euractiv.com/section/politics/news/domestic-violence-increases-in-france-during-covid-19-lockdown/](NULL) - Cullen R. As domestic abuse cases rise, France looks for new solutions to help victims [Internet]. CGTN. [cited 2020 Jun 15]. Available from: [https://newseu.cgtn.com/news/2020-04-29/How-France-is-tackling-the-rise-in-domestic-abuse-cases-Q42SkrHPgY/index.html](NULL) - French gender equality minister on helping victims of domestic violence during lockdown [Internet]. France24. [cited 2020 Jun 11]. Available from: [https://www.france24.com/en/20200421-french-gender-equality-minister-on-helping-victims-of-domestic-violence-during-lockdown](NULL) - Abels R. Als een Française om ‘masker 19’ vraagt, weet de apotheker dat hij de politie moet bellen. Trouw [Internet]. Available from: [https://www.trouw.nl/buitenland/als-een-francaise-om-masker-19-vraagt-weet-de-apotheker-dat-hij-de-politie-moet-bellen~b70df617/?referer=https%3A%2F%2Fen.wikipedia.org%2F](NULL) - Talmazan Y, Sirna L, Munoz Ratto H, Ing N. European countries develop new ways to tackle domestic violence during coronavirus lockdowns [Internet]. NBC news. [cited 2020 Jun 16]. Available from: [https://www.nbcnews.com/news/world/european-countries-develop-new-ways-tackle-domestic-violence-during-coronavirus-n1174301](NULL) - Cullen R. As domestic abuse cases rise, France looks for new solutions to help victims [Internet]. CGTN. [cited 2020 Jun 15]. Available from: [https://newseu.cgtn.com/news/2020-04-29/How-France-is-tackling-the-rise-in-domestic-abuse-cases-Q42SkrHPgY/index.html](NULL) |
| **Germany**   - Steinert J, Ebert C. Domestic violence during the coronavirus pandemic. TUM. - Ausnahmesituation für Familien [Internet]. Die Bundesregierung. [cited 2020 Jun 15]. Available from: [https://www.bundesregierung.de/breg-de/themen/coronavirus/ausnahmesituation-fuer-familien-1734472](NULL) - Schindler F. „Das eigene Zuhause ist für viele Frauen kein sicherer Ort“ [Internet]. Welt. [cited 2020 Jun 15]. Available from: [https://www.welt.de/politik/deutschland/article206709651/Ausgangssperre-wegen-Corona-Fuer-Frauen-eine-bedrohliche-Situation.html](NULL) |
| **Ireland**   - Leahy P, Cullen P, Lunch S, Kelly F. Coronavirus: Schools, colleges and childcare facilities in Ireland to shut [Internet]. The Irish Times. [cited 2020 Jun 19]. Available from: [https://www.irishtimes.com/news/health/coronavirus-schools-colleges-and-childcare-facilities-in-ireland-to-shut-1.4200977](NULL) - Public health measures in place right now [Internet]. Gov.ie. [cited 2020 Jun 19]. Available from: [https://www.gov.ie/en/publication/cf9b0d-new-public-health-measures-effective-now-to-prevent-further-spread-o/?referrer=/en/publication/539d23-stay-at-home-the-latest-public-health-measures-to-prevent-the-spread/](NULL) - Media Release: 39% increase in calls answered by Women’s Aid during Covid-19 Lockdown. [Internet]. Women’s Aid. [cited 2020 Jun 17]. Available from: [https://www.womensaid.ie/about/newsevents/news/2020/05/29/media-release-39-increase-in-calls-answered-by-wom/](NULL) - Minister Doherty Eases Access to Rent Supplement for Victims of Domestic Violence [Internet]. Protection, Department of Employment Affairs and Social. [cited 2020 Jun 17]. Available from: [https://www.gov.ie/en/press-release/44b3b-minister-doherty-eases-access-to-rent-supplement-for-victims-of-domestic-violence/](NULL) - Operation Faoiseamh sees 107 prosecutions in relation to domestic abuse [Internet]. Irish Examiner. [cited 2020 Jun 17]. Available from: [https://www.irishexaminer.com/breakingnews/ireland/operation-faoiseamh-sees-107-prosecutions-in-relation-to-domestic-abuse-1004143.html](NULL) - ]. Available from: [https://www.irishexaminer.com/breakingnews/ireland/operation-faoiseamh-sees-107-prosecutions-in-relation-to-domestic-abuse-1004143.html](NULL) - 36. Flanagan C. Speech by Minister for Justice and Equality Charlie Flanagan TD on Community Policing in the context of COVID-19 [Internet]. Department of Justice and Equality. [cited 2020 Jun 17]. Available from: [https://www.gov.ie/en/speech/907ed-speech-by-minister-for-justice-and-equality-charlie-flanagan-td-on-community-policing-in-the-context-of-covid-19/](NULL) - Still Here Campaign [Internet]. Department of Justice and Equality. [cited 2020 Jun 17]. Available from: [https://www.stillhere.ie/awareness-campaign/](NULL) - Minister Doherty Eases Access to Rent Supplement for Victims of Domestic Violence [Internet]. Protection, Department of Employment Affairs and Social. [cited 2020 Jun 17]. Available from: [https://www.gov.ie/en/press-release/44b3b-minister-doherty-eases-access-to-rent-supplement-for-victims-of-domestic-violence/](NULL) |
| **Italy**   - Coronavirus: Northern Italy quarantines 16 million people [Internet]. BBC news. [cited 2020 Jun 19]. Available from: [https://www.bbc.com/news/world-middle-east-51787238](NULL) - Donato V Di, Reynolds E, Picheta R. All of Italy is in lockdown as coronavirus cases rise [Internet]. CNN. [cited 2020 Jun 19]. Available from: [https://edition.cnn.com/2020/03/09/europe/coronavirus-italy-lockdown-intl/index.html](NULL) - Inchiesta DI, Pietro D. Commissione parlamentare di inchiesta. 1995;2020:84–6. - Cristoferi C, Fonte G. In Italy, support groups fear lockdown is silencing domestic abuse victims [Internet]. The Jakarta Post. [cited 2020 Jun 11]. Available from: [https://www.thejakartapost.com/news/2020/04/06/in-italy-support-groups-fear-lockdown-is-silencing-domestic-abuse-victims.html](NULL) - Ruggieri R. Violenza domestica ai tempi del Coronavirus: le misure per contrastarla [Internet]. Reti di Giustizia. [cited 2020 Jun 6]. Available from: [https://www.avvocatirandogurrieri.it/leggi-e-diritto/violenza-domestica-ai-tempi-del-coronavirus-le-misure-per-contrastarla](NULL) - Misure Covid-19 e violenza sulle donne \| Numeri utili e App [Internet]. Universita degli Studie di Ferrara. [cited 2020 Jun 20]. Available from: [http://www.unife.it/it/covid19/uniferestaonlineturestaacasa/covid-19-e-violenza-sulle-donne](NULL) - Dentamaro B. Covid-19, violenza domestica e risposta internazionale [Internet]. Piueuropa. [cited 2020 Jun 20]. Available from: [https://piueuropa.eu/2020/04/22/covid-19-violenza-domestica-e-risposta-internazionale/#](NULL)! - UNDP. Gender-based violence and Covid-19. 2020; |
| **Portugal**   - Demony C, Waldersee V. Portugal extends coronavirus lockdown, promises masks and hand gel [Internet]. Reuters. [cited 2020 Jun 19]. Available from: [https://www.reuters.com/article/us-health-coronavirus-portugal/portugal-extends-coronavirus-lockdown-promises-masks-and-hand-gel-idUSKCN21Y2FR](NULL) - Afinal, violência doméstica não aumentou 50% devido ao coronavírus: desceu 26%. Crimes de burla é que aumentaram [Internet]. Observador. [cited 2020 Jun 18]. Available from: [https://observador.pt/2020/04/08/covid-19-devido-a-quarentena-violencia-domestica-aumentou-50-no-mes-de-marco/](NULL) - Governo destaca medidas de combate às desigualdades em cenário de pandemia [Internet]. Repuclica Portuguesa. [cited 2020 Jun 18]. Available from: [https://www.portugal.gov.pt/pt/gc22/comunicacao/noticia?i=governo-destaca-medidas-de-combate-as-desigualdades-em-cenario-de-pandemia](NULL) - Portuguesa R. Intervenção da Ministra de Estado e da Presidência Audição parlamentar regimental na 1^a^ Comissão, 13 de maio de 2020 [Internet]. Available from: [https://www.portugal.gov.pt/download-ficheiros/ficheiro.aspx?v=baaab247-f06f-4785-8831-f129874f07ec](NULL) - Segurança C-. NOTA À COMUNICAÇÃO SOCIAL. 2020; |
| **Spain**   - Jones S. Spain orders nationwide lockdown to battle coronavirus [Internet]. The Guardian. [cited 2020 Jun 19]. Available from: [https://www.theguardian.com/world/2020/mar/14/spain-government-set-to-order-nationwide-coronavirus-lockdown](NULL) - Jones S. Spanish town faces police lockdown to contain coronavirus [Internet]. The Guardian. [cited 2020 Jun 19]. Available from: [https://www.theguardian.com/world/2020/mar/07/spanish-town-faces-police-lockdown-to-contain-coronavirus](NULL) - Burgen S. Women killed in Spain as coronavirus lockdown sees rise in domestic violence [Internet]. The Guardian. [cited 2020 Jun 13]. Available from: [https://www.theguardian.com/global-development/2020/apr/28/three-women-killed-in-spain-as-coronavirus-lockdown-sees-rise-in-domestic-violence](NULL) - Equality OF. Executive Summary: contingency plan to combat gender violence during the Covid-19 crisis. 2020;(April). - Rosco M. “Mascarilla-19” la palabra clave que pueden usar las víctimas de violencia de género en las farmacias [Internet]. Lahoradigital. [cited 2020 Jun 20]. Available from: [https://lahoradigital.com/noticia/26266/sanidad/mascarilla-19-la-palabra-clave-que-pueden-usar-las-victimas-de-violencia-de-genero-en-las-farmacias.html](NULL) |
| **Switzerland**   - New coronavirus: Measures, ordinance and explanations [Internet]. FOPH. [cited 2020 Jun 19]. Available from: [https://www.bag.admin.ch/bag/en/home/krankheiten/ausbrueche-epidemien-pandemien/aktuelle-ausbrueche-epidemien/novel-cov/massnahmen-des-bundes.html#-406263106](NULL#-406263106) - Oertli B. Meldungen zu häuslicher Gewalt haben nicht zugenommen [Internet]. SRF. [cited 2020 Jun 16]. Available from: [https://www.srf.ch/news/schweiz/trotz-corona-isolation-meldungen-zu-haeuslicher-gewalt-haben-nicht-zugenommen](NULL) - Anzahl Fälle der häuslichen Gewalt bleibt stabil – auch in der Corona-Krise [Internet]. Bereich Allgemein. [cited 2020 Jun 16]. Available from: [https://www.sg.ch/news/sgch_allgemein/2020/05/anzahl-faelle-der-haeuslichen-gewalt-bleibt-stabil---auch-in-der.html](NULL) - Häusliche Gewalt während Corona: Situation in den meisten Kantonen stabil [Internet]. EBG. [cited 2020 Jun 16]. Available from: [https://www.ebg.admin.ch/ebg/de/home/themen/haeusliche-gewalt/koordination-und-vernetzung.html](NULL) - Häusliche Gewalt in den meisten Kantonen nicht angestiegen [Internet]. Pilatus Today. [cited 2020 Jun 16]. Available from: [https://www.pilatustoday.ch/schweiz/haeusliche-gewalt-in-den-meisten-kantonen-nicht-angestiegen-138079929](NULL) - Schutz vor häuslicher Gewalt in Corona-Zeiten: Bund beruft Taskforce ein [Internet]. EBG. [cited 2020 Jun 16]. Available from: [https://www.ebg.admin.ch/ebg/de/home/themen/haeusliche-gewalt/koordination-und-vernetzung.html](NULL) - Anzahl Fälle der häuslichen Gewalt bleibt stabil – auch in der Corona-Krise [Internet]. Bereich Allgemein. [cited 2020 Jun 16]. Available from: [https://www.sg.ch/news/sgch_allgemein/2020/05/anzahl-faelle-der-haeuslichen-gewalt-bleibt-stabil---auch-in-der.html](NULL) |
| **The Netherlands**   - Premier Rutte: dit is een intelligente lockdown [Internet]. NOS. [cited 2020 Jun 19]. Available from: [https://nos.nl/video/2328097-premier-rutte-dit-is-een-intelligente-lockdown.html](NULL) - Meer hulpvragen huiselijk geweld via online chatdiensten [Internet]. Zembla BNNVARA. [cited 2020 Jun 11]. Available from: [https://www.bnnvara.nl/zembla/artikelen/meer-hulpvragen-huiselijk-geweld-via-online-chatdiensten](NULL) - Ministerie van Volksgezondheid, Welzijn en sport. R. Wees alert op de thuissituatie. May 18, 2020. - Hoorn O. Chatfunctie ingevoerd bij Veilig Thuis [Internet]. GGD GHOR. [cited 2020 Jun 20]. Available from: [https://www.ggdghorkennisnet.nl/thema/veilig-thuis/nieuws/14726-chatfunctie-ingevoerd-bij-veilig-thuis](NULL) - Huiselijk geweld melden bij apotheek via codewoord [Internet]. KNMP. [cited 2020 Jun 11]. Available from: [https://www.knmp.nl/actueel/nieuws/nieuws-2020/huiselijk-geweld-melden-bij-apotheek-via-codewoord](NULL) |
| **United Kingdom**   - Boris Johnson’s address to the nation in full [Internet]. The Guardian. [cited 2020 Jun 19]. Available from: [https://www.theguardian.com/uk-news/2020/mar/23/boris-johnsons-address-to-the-nation-in-full](NULL) - Ivana K, Di Donato V. Woman are using code words at pharmacies to escape domestic violence [Internet]. CNN. [cited 2020 Jun 11]. Available from: [https://edition.cnn.com/2020/04/02/europe/domestic-violence-coronavirus-lockdown-intl/index.html](NULL) - Home Office preparedness for Covid-19 (Coronavirus): domestic abuse and risks of harm within the home [Internet]. [cited 2020 Jun 12]. Available from: [https://publications.parliament.uk/pa/cm5801/cmselect/cmhaff/321/32105.htm](NULL) - UK Government. Home Secretary’s statement on domestic abuse and coronavirus (COVID-19)[internet]. Accessed june 11, 2020 Available from [https://www.gov.uk/government/speeches/home-secretary-outlines-support-for-domestic-abuse-victims](NULL) - Prospectus B. Domestic Abuse Safe Accommodation : MHCLG COVID-19 Emergency Support Fund Bid Prospectus. 2020; |
